# A Novel Tyrosine Kinase Axis in Innate Immune Signaling

**DOI:** 10.3390/v18010010

**Published:** 2025-12-20

**Authors:** Santanu Das, Pracheta Sengupta, Manoj Veleeparambil, Saurabh Chattopadhyay

**Affiliations:** Department of Microbiology, Immunology, and Molecular Genetics, University of Kentucky College of Medicine, Lexington, KY 40536, USAmmve226@uky.edu (M.V.)

**Keywords:** tyrosine kinase, Lyn, Syk, TLR9, STING

## Abstract

Tyrosine phosphorylation has emerged as a central regulatory mechanism in innate immunity. Building on our recent studies that Syk and EGFR sequentially phosphorylate TLR9 to fully activate it, we discuss how similar mechanisms operate across other Toll-like receptors and the cytosolic DNA sensor STING. Evidence from complementary systems reveals that receptor and nonreceptor tyrosine kinases, including Src-family kinases, Syk, BTK, and EGFR, form an integrated signaling network that triggers receptor activation, trafficking, and downstream gene expression. Scavenger receptors such as SR-A further drive this kinase cascade by coordinating viral recognition to TLR activation. These observations reveal a novel ‘tyrosine kinase axis’ that connects nucleic acid sensing to spatially controlled innate immune signaling and highlight new opportunities to modulate innate immunity through tyrosine kinase regulation.

## 1. Introduction

**Figure 1:**
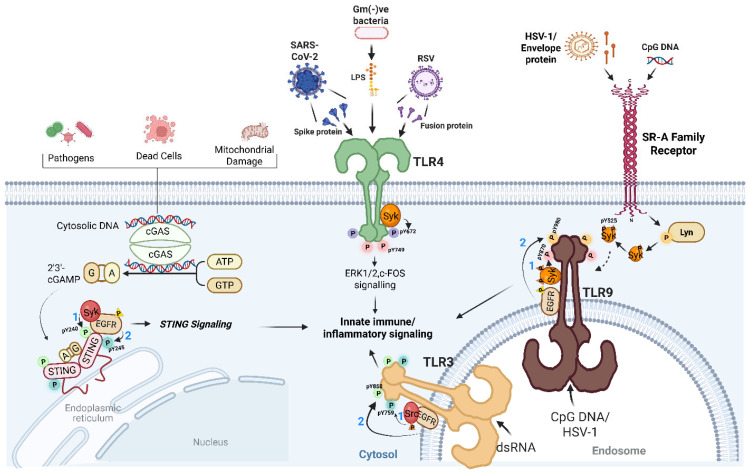
Tyrosine kinase-driven regulation of antiviral innate immune sensing. Toll-like receptors (TLRs) located at the cell surface or on endosomal membranes require ligand-induced tyrosine phosphorylation on their cytoplasmic domains to activate intracellular signaling. Multiple tyrosine kinases, including Src-family kinases, Syk, and EGFR, phosphorylate specific TLR residues to regulate receptor activation, adaptor recruitment, and cytokine production. Syk and EGFR also phosphorylate STING, facilitating its trafficking and IRF3 activation during cytosolic DNA sensing. Our findings further identify scavenger receptors (SR-A) as upstream regulators that prime TLR9 activation by initiating a Lyn-Syk kinase cascade. The tyrosine kinase axis can also either directly or indirectly activate the Ser/Thr kinases to initiate the intracellular signaling pathway. Numbered steps indicate key biochemical events within these regulated kinase cascades.

Innate immune sensors are the front line of antiviral defense, sensing pathogen-associated molecular patterns (PAMPs) to activate intracellular antiviral and inflammatory programs [1,2,3,4,5]. We recently revealed a crucial and previously unrecognized feature of this dogma: ligand-induced sequential tyrosine phosphorylation as a molecular switch in Toll-like receptor 9 (TLR9) activation [6]. We found that two tyrosine kinases, Syk and EGFR, sequentially phosphorylate TLR9 on Y870 and Y980, respectively, to trigger adaptor recruitment and the induction of downstream target genes. This stepwise process, initiated by scavenger receptor A (SR-A)-mediated activation of Lyn, a nonreceptor tyrosine kinase, establishes a regulated kinase cascade that ensures complete TLR9 activation. Our findings further suggest a phosphotyrosine-based checkpoint of TLR9 signaling during microbial DNA sensing (Figure 1).

A growing body of evidence indicates that tyrosine phosphorylation is a common mechanism across multiple antiviral TLRs [7]. We showed that TLR3 requires Src and EGFR-dependent phosphorylation of its cytoplasmic domain, facilitating TRIF recruitment and IRF3 activation [8,9]. TLR4 signaling, in contrast, depends on EGFR for the IRF3-driven antiviral arm, but not for NF-κB-mediated inflammatory responses [10]. These studies suggest that EGFR acts as a nodal kinase, connecting diverse TLRs to specific transcriptional outcomes. Together, we uncover, through this series of studies, an emerging theme: tyrosine kinases function not only as regulators but also as activators that couple ligand sensing with receptor phosphorylation and downstream transcriptional programs. The phosphotyrosine-mediated regulation extends beyond TLRs to cytosolic DNA sensing by STING. STING activation, like TLR9, also requires sequential tyrosine phosphorylation of specific residues (Y240, Y245) by Syk and EGFR, which controls its trafficking from the ER to endosomes [11]. These modifications enable IRF3 activation and interferon (IFN) induction, underscoring how tyrosine phosphorylation integrates receptor activation with intracellular localization [12,13]. These findings, together with TLRs and STING, define a shared tyrosine kinase-dependent emerging paradigm that dictates the strength and localization of signaling responses.

Upstream of these signaling events, our studies revealed an unexpected but essential role of scavenger receptors (SRs) in activating a cascade of non-receptor tyrosine kinases. SR-A, a member of the SR family, acts as an initiating receptor that recognizes CpG DNA and triggers Lyn- and Syk-mediated activation, prior to and independent of, TLR9 signaling [6,14,15]. Other SRs, e.g., MARCO and SR-B1, also co-activate the TLRs during viral entry or immune activation, suggesting that these cell surface receptors may function as kinase-activating proteins for the immune sensors [16,17]. The interplay between SRs, tyrosine kinases, and innate immune sensors dictate the strength, timing, and localization of innate immune signaling [18]. These observations point toward a “tyrosine–kinase axis” that coordinates microbial sensing from the plasma membrane to the endosome and cytosol. The convergence of EGFR, Syk, and Src-family kinases across TLRs and STING defines a unifying theme that ensures efficient antimicrobial programs while regulating aberrant inflammation [11,19]. Understanding how this cascade is spatially and temporally regulated may reveal new strategies to modulate host defenses against microbial infections or to temper pathological inflammation where these pathways are dysregulated.

## 2. Tyrosine Kinases as Central Orchestrators of Antiviral TLR Signaling

Viral nucleic acids are potent activators of TLRs, triggering type I interferon (IFN) and proinflammatory cytokine production through distinct adaptor-mediated signaling cascades. These pathways are critically dependent on tyrosine phosphorylation events that dictate receptor activation and downstream signaling. Following viral recognition, receptor and non-receptor tyrosine kinases are rapidly activated to participate in antiviral signaling. Among the earliest to act are Src-family kinases (Lyn, Fyn, Hck), which phosphorylate TLR cytoplasmic domains and associated adaptor proteins to activate downstream signaling [7,20]. Bruton’s tyrosine kinase (BTK) serves as a critical kinase, enabling the TLR–adaptor complex formation, promoting cytokine production, and shaping the magnitude of inflammatory responses during viral infection [7,21,22]. Syk, a key cytoplasmic tyrosine kinase, plays an indispensable role in TLR2- and TLR4-mediated inflammatory responses, driving the phosphorylation events that promote NF-κB and MAPK activation [21]. Site-specific tyrosine phosphorylation, e.g., Y672 and Y749 of TLR4, triggers ERK activation and thereby fine-tunes cytokine induction [23,24].

The emerging role of EGFR as a noncanonical yet essential kinase in innate antiviral signaling is an unexpected yet most striking feature. EGFR phosphorylates TLR3 and TLR9 directly, controlling the key early steps of receptor activation [9,25]. Viral nucleic acids that engage these endosomal TLRs activate EGFR, which in turn phosphorylates conserved tyrosine residues on TLR cytoplasmic tails. In TLR9, phosphorylation of Y980 is indispensable for MyD88 recruitment and cytokine induction. Mutation of this site or pharmacological inhibition of EGFR diminishes the cytokine response, underscoring its role as a tyrosine kinase that facilitates innate immune sensors [6]. EGFR is also involved in the phosphorylation of TLR7 in macrophages, regulating glomerular injury [26]. EGFR, therefore, acts as a molecular bridge that couples viral recognition to receptor activation, often compensating for canonical immune signaling kinases. Tyrosine phosphorylation also provides a platform for the recruitment of phosphatidylinositol 3-kinase (PI3K) and other downstream effectors, integrating tyrosine kinase signaling with serine/threonine kinase cascades [10]. This interplay between tyrosine and serine/threonine kinases, either directly or indirectly, controls TLR signaling intensity, duration, and specificity and prevents aberrant inflammation (Figure 1). Finally, whether epidermal growth factor (EGF), the cognate ligand for EGFR, can broadly potentiate PAMP- or DAMP-driven TLR signaling remains largely unexplored. EGF stimulation, however, can enhance dsRNA-induced TLR3 activation [9], providing precedent that growth factor-mediated EGFR activation can amplify innate immune receptor function. Noncanonical EGFR activation can activate TBK1 and IRF3 signaling pathways in cancer cells [27]. Involvement of innate immune sensors in this signaling pathway remains to be identified.

## 3. Tyrosine Phosphorylation Extends to STING Signaling

The theme of tyrosine phosphorylation-mediated control of TLR signaling extends to the STING signaling pathway. Following viral infection, Syk phosphorylates STING at Y240, enabling its exit from the endoplasmic reticulum and initiating the antiviral IFN response. Subsequently, EGFR phosphorylates STING at Y245, facilitating its endosomal translocation and ensuring productive activation of IRF3 (Figure 1) [11,12,28]. Conversely, phosphatases PTPN1 and PTPN2 attenuate STING activation by dephosphorylating these sites, temporally regulating signaling outcomes [29]. Additional evidence implicates Src-family kinases and PTK2B in regulating STING oligomerization and TBK1 recruitment, while BTK can phosphorylate upstream DNA sensors such as DDX41, further enhancing STING activation [30,31]. Together, these findings reveal that tyrosine phosphorylation is essential for both spatial and temporal control of STING functions. Like in the TLR system, these phosphorylation events also provide a checkpoint of STING activation, trafficking and signalosome assembly. This conserved mechanism across TLRs and STING strengthens the existence of a shared ‘tyrosine kinase axis’ in antiviral innate immunity (Table 1).

## 4. Scavenger Receptors: Gatekeepers of the Tyrosine Kinase Axis

A key upstream layer in this tyrosine phosphorylation-regulated network involves scavenger receptors (SRs), which act as both pathogen sensors and co-receptors that coordinate the kinase cascades [39,40,41]. SRs can bind viral components directly or associate with TLRs to enhance ligand uptake and signaling specificity [42]. SR-A primes TLR9 signaling during CpG DNA sensing and serves as an initiating receptor that activates Lyn and Syk tyrosine kinase axis, prior to and independent of, TLR engagement [6]. SR-A also allows effective priming of the kinase cascade required for downstream tyrosine phosphorylation of TLR9. Other SRs, including MARCO and SR-B1, have multifaceted roles in viral infection, either facilitating viral entry or modulating innate immune responses [17,43,44]. SR-B1, for example, cooperates with ACE2 during SARS-CoV-2 entry [45] and associates with ApoA-1 to promote dengue virus infection [46], while SR-A1 and SERC-1 interact with TLR2 in dendritic cells to drive adaptive immune responses [47]. Beyond serving as viral entry facilitators, these receptors activate the tyrosine kinase axis and coordinate it with the TLRs. Their ability to recognize ligands and engage Src-family kinases suggests that SRs are active participants in setting the tone of tyrosine phosphorylation in antiviral signaling. By bridging extracellular recognition and intracellular kinase activation, SRs ensure that TLR tyrosine phosphorylation occurs in the appropriate context and cellular compartment.

## 5. Conclusion: An Emerging ‘Tyrosine Kinase Axis’ Theme in Antiviral Innate Immunity

Evidence from TLRs, STING, and scavenger receptors points to a unifying theme: tyrosine phosphorylation acts as a conserved molecular switch that orchestrates antiviral signaling across innate immune receptors. This tyrosine kinase axis, comprising Src-family kinases, Syk, BTK, and EGFR, controls receptor activation, subcellular trafficking, adaptor assembly, and transcriptional outcome of infected and damaged cells. Whether the tyrosine kinase axis is ubiquitously activated during viral infection and whether viruses can antagonize it remain to be investigated. Furthermore, the requirement for SR-A by TLRs has not been fully evaluated; extensive screening of its roles in other TLR signaling pathways will be conducted in the future. By coupling viral sensing with kinase activation, the system provides signal specificity and prevents undesired inflammation. Future studies will be needed to map these interactions and define which kinases act at which stage, how their activities are compartmentalized, and how phosphatases reset the signaling outcomes. Emerging new technologies, e.g., high-resolution imaging, proximity labeling-coupled proteomics, and spatial transcriptomics, within defined subcellular microenvironments, will help resolve these questions. Leveraging these approaches will delineate how the tyrosine kinase axis is dynamically assembled, compartmentalized, and rewired during viral infection and inflammatory stimulation. Understanding this dynamic equilibrium between kinases and phosphatases through high-throughput drug screens will not only deepen our insights into antiviral innate immunity but also offer new therapeutic strategies to fine-tune host responses in infection, inflammation, and autoimmunity.

## Figures and Tables

**Table 1:** Specific members of the novel tyrosine kinase axis involved in innate immune signaling.

Innate Immune Sensors	Ligands and Stimuli	Tyrosine Kinases Involved	References
TLR2	Lipoproteins, Peptidoglycans, Pam3CSK4	Fyn, Btk, Lyn	[21,32,33]
TLR3	dsRNA, Rabies, HSV-1	Src, EGFR, Btk	[9,22,34]
TLR4	LPS, SARS-CoV-2, RSV	EGFR, Syk, Lyn, Hck	[10,20,23,35,36]
TLR7	R848, SARS-CoV-2, HSV-1	EGFR	[26,37,38]
TLR9	CpG DNA, HSV-1	Syk, EGFR, Lyn	[6,7,25]
cGAS–STING	Cytosolic DNA, HSV-1, cytomegalovirus	Syk, EGFR	[11,12,31]

## Data Availability

No new data were created or analyzed in this study.
